# Copy Number Variations in Genetic Diagnosis of Congenital Adrenal Hyperplasia Children

**DOI:** 10.3389/fgene.2022.785570

**Published:** 2022-03-02

**Authors:** Aisha Tolba, Iman Mandour, Noha Musa, Fatma Elmougy, Mona Hafez, Sahar Abdelatty, Amany Ibrahim, Hend Soliman, Bahaaeldin Labib, Yasmine Elshiwy, Tarek Ramzy, Marwa Elsharkawy

**Affiliations:** ^1^ Clinical and Chemical Pathology Department, Cairo University, Giza, Egypt; ^2^ Diabetes, Endocrine and Metabolism Pediatric Unit, Cairo University, Giza, Egypt; ^3^ Royal College of Surgeons in Ireland, Medical University of Bahrain, Giza, Egypt

**Keywords:** 21-Hydroxylase deficiency, CYP21A2, multiplex ligation-dependent probe amplification, copy number variations, congenital adrenal hyperplasia

## Abstract

**Background:** Congenital adrenal hyperplasia (CAH) is a monogenic disorder caused by genetic diversity in the *CYP21A2* gene, with 21-hydroxylase deficiency (21-OHD) as the most common type. Early sex assignment and early diagnosis of different genetic variations with a proper technique are important to reduce mortality and morbidity. Proper early sex identification reduces emotional, social, and psychological stress.

**Aim:** Detection of a spectrum of aberrations in the *CYP21A2* gene, including copy number variations, gene conversion, chimeric genes, and point variations.

**Methods:** The *CYP21A2* gene was screened using MLPA assay in 112 unrelated Egyptian children with 21-OHD CAH (33 males and 79 females).

**Results:** In the studied group, 79.5% were diagnosed within the first month of life. 46.8% of the genetic females were misdiagnosed as males. Among the copy number variation results, large deletions in 15.4% and three types of chimeric genes in 9% (CH-1, CH-7, and CAH-X CH-1) were detected. Regarding gene dosage, one copy of *CYP21A2* was found in 5 cases (4.5%), three copies were detected in 7 cases (6.3%), and one case (0.9%) showed four copies. Eight common genetic variants were identified, I2G, large deletions, large gene conversion (LGC), I172N, F306 + T, -113 SNP, 8bp Del, and exon 6 cluster (V237E and M239K) with an allelic frequency of 32.62%, 15.45%, 7.30%, 3.00%, 2.58%, 2.15%, 0.86%, and 0.86%, respectively.

**Conclusion:** High prevalence of copy number variations highlights the added value of using MLPA in routine laboratory diagnosis of CAH patients.

## Introduction

Congenital adrenal hyperplasia (CAH) represents a group of common defects in the adrenal gland steroidogenesis; the main form is 21-hydroxylase enzyme deficiency (21-OHD; OMIM #201910) (Chan et al., 2011). Patients with the classic phenotype can present with life-threatening salt-wasting (SW) and simple virilizing (SV) types depending on the enzymatic activity of the 21-OH enzyme (Rabbani et al., 2011). There is a higher prevalence of CAH in Egypt (1 in 1,209 live births) and Arab countries due to a higher rate of consanguinity (Temtamy and Aglan, 2012) (Tayel et al., 2011) (Alshabab et al., 2015).

In males, CAH is a medical emergency, with an urgent need for early diagnosis as it can be fatal in cases with SW-CAH. In females, CAH-SV can also be considered a social emergency (Kutney et al., 2016). The sex of a newborn is usually determined at birth on the basis of external genital appearance. Thus, children with atypical genitalia frequently require reassignment of sex because of incorrect original identification. As sex is a fundamental attribute of human life, its reversal after the original assignment is usually associated with marked emotional, social, and psychological stress with accompanying shame (Wisniewski, 2017).

First-line diagnostic tests for CAH include karyotype analysis, hormonal evaluation (from 48 h after birth) for 17-hydroxyprogesterone (17OHP), androgens, basal cortisol, and adrenocorticotropic hormone (ACTH), and ultrasound (for detecting Mullerian structures). The definitive diagnosis of 21OHD CAH is through the assessment of serum 17OHP levels (levels above 10 ng/dl are diagnostic). Additional imaging tests may be recommended, including genogram, retrograde urethrogram, or cystoscopy/vaginoscopy (in females with atypical genitalia). When the gonads are not detected by ultrasound, magnetic resonance imaging is indicated. Molecular studies are performed to check for gene mutations (Guerrero-Fernández et al., 2018).

Management of CAH cases includes lifelong hormonal therapy and reconstructive surgery for female cases with atypical genitalia (feminizing genitoplasty) (Savanelli et al., 2008). This is best done in early infancy to confer an early physical appearance consistent with the gender/sex of rearing and to cause less psychological trauma than if delayed (Speiser et al., 2018; Musa et al., 2020).

While CAH-21OH is a monogenic disorder, the human 21-OH gene (cytochrome P450 family 21 subfamily A member 2, CYP21A2) encodes the 21-OH enzyme, and a highly homologous inactive pseudogene, CYP21A1P, is located closely adjacent to the CYP21A2 gene, approximately 30 kb apart in tandem arrangement with the C4A and C4B genes (Blaskó et al., 2009; Choi et al., 2016; Daae et al., 2018). These arrangements comprise the most frequent bimodular RCCX of the RP1-C4-CYP21A1P-TNXA-RP2-C4-CYP21A2-TNXB gene sequence in 69% of alleles in Caucasians (Lee et al., 2010; Tsai et al., 2012). Recombination events commonly occur when a loop forms in the linear DNA of the chromosome. The active CYP21A2 and inactive CYP21A1P genes lie against each other, allowing the transfer of genetic material between them. The advantage of this physiological event is that it increases immunological diversity over time. However, the CYP21A2 gene sits adjacent to the HLA locus and becomes a passive participant in this process (Huynh et al., 2009).

The high degree of sequence similarity between active and pseudogene allows two types of intergenic recombination events that are responsible for about 95% of the mutations associated with 21-OHD (Vrzalová et al., 2011). The first is gene conversion events: approximately 65%–70% of the deleterious mutations are derived from pseudogene CYP21A1P due to small gene conversions, including I2G (28%), I172N (9%), V281L (9%), Q318X (4%), R356W (4%), E6 cluster [I235N, V236E, and M238K] (4%), 8bp Del (3%), P30L (2%), and F306 + T (1%) (Xu et al., 2013). If these transfers comprise a large region, it is called a “large-scale conversion” (Toraman et al., 2013).

When targeted mutation analysis detects multiple mutations, it is possible that the mutations are most likely arising from gene conversion (Nimkarn et al., 1993). The second is unequal crossing over during meiosis: the remaining 20%–25% of the intergenic recombination is represented by CYP21A2 gene deletions or CYP21A1P/CYP21A2 chimeric genes (Concolino et al., 2009). Nine different CYP21A1P/CYP21A2 chimeric genes were described according to their breakpoints (CH-1 to CH-9) (Chen et al., 2012; Veldhuisen et al., 2015). The third common variation in CYP21A2 is spontaneous point mutations (Xu et al., 2013).

Multiplex Ligation-dependent Probe Amplification (MLPA) assay with the addition of common point-mutation (PMS) MLPA probes represents the gold standard in CAH diagnosis and a sensitive tool for identification of chimerical genes (Choi et al., 2016) (Erlandson et al., 2008).

The aim of this study is to perform genetic analysis of CAH 21-OHD Egyptian children to detect a spectrum of aberrations in the CYP21A2 gene, including copy number variations, gene conversion, gene deletion, chimeric genes, and point variations using MLPA analysis.

## Materials and Methods

Editorial policies and ethical considerations: children with CAH were recruited from the Diabetes Endocrine Metabolism Pediatric Unit (DEMPU), Children’s Hospital, Cairo University, after obtaining written informed consent from all participants or their legal guardians. The study was approved by the Research Ethics Committee of Cairo University. The diagnosis of CAH due to 21-OHD (salt wasting and simple virilizing) was based on both clinical and biochemical examinations (elevated 17-hydroxyprogesterone).

Three millilitres of blood were collected from each subject in a sterile EDTA vacutainer for the genotyping technique. DNA was extracted using the DNA extraction kit QIAamp (DNA Blood Mini Kit) supplied by QIAGEN (Thermo Fisher Scientific, USA) according to the manufacturer’s instructions. Then Selective Adaptor Ligation, Selective Amplification (SALSA) MLPA Probemix P050-C1 CAH (Catalog no. P050-100R) in combination with the SALSA MLPA EK1 reagent kit (EK1-FAM) supplied by MRC-Holland was used.

Multiplex ligation-dependent probe amplification analysis of the CYP21A2 gene included the following steps according to the manufacturer’s instructions: DNA denaturation, hybridization reaction, ligation reaction, PCR reaction, and fragment separation by capillary electrophoresis, ABI 3500 Genetic Analyzer (Applied Biosystems, Thermo Fisher Scientific, USA).

### Bioinformatics and Statistical Analysis

The raw data were analysed using Coffalyser.net software, the MLPA analysis tool developed at MRC-Holland. It was used for data analysis in combination with the appropriate lot-specific MLPA Coffalyser sheet. They were available on www.mlpa.com.

The nomenclature of the variants reported in the current study are according to the Human Genome Variation Society (HGVS) (denDunnen et al., 2016) as follows (NM_000500.8): 113 SNP (rs1246774295, c.-113G > A), I2G (rs6467, c.293–13C > G), 8bp Del (rs387906510, c.332_339delGAGACTAC, c.332_339del), I172N (rs6475, c.518T > A), Exon 6 cluster variants [V237E (rs12530380, c.713T > A), M239K (rs6476), c.719T > A], and F306 + T (rs267606756, c.923dup).

Data were analysed using IBM SPSS Advanced Statistics version 21.0 (IBM Corp., Armonk, NY). Qualitative data were expressed as frequency and percentage.

## Results

One hundred and twelve probands with 21-OHD were recruited for the study. The majority (79.5%, 89/122) were diagnosed in the first month of life. 50/112 (44.6%) were diagnosed at birth (day 0) and 39/112 (34.8%) between1–30 days. Seventy-six (67.9%) patients were born to consanguineous parents. By karyotyping, the genetically confirmed females constituted 79/112 (70.5%) and 33/112 (29.5%) were males. The female-to-male ratio was 2.4:1, and 37/79 (46.8%) of genetic females were misdiagnosed as males.

The majority of females presented with ambiguous genitalia (78/79, 98.7%). In most cases, 49/79 (62%) presented with Prader stage III, followed by stage IV (16.5%). The remaining cases presented with Prader stage II (10.1%), I (7.6%), and V (2.5%), while 4/33 males (12.1%) presented with precocious puberty.

**FIGURE 1:**
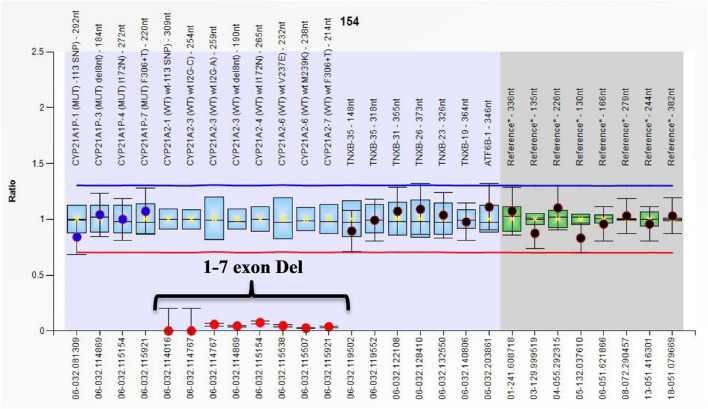
Ratio chart showing the homozygous 1–7 exon Del CYP21A2 gene.

All cases in the current study had the classical form of 21-OHD. Eighty-eight out of 112 patients (78.6%) were SW, and 24/112 (21.4%) cases had the SV form. The phenotype of all cases with large gene conversions and/or deletions is SW except for one case that was SV (LGC 1-3 exon on one allele and Del 1-7 exon Del on the other allele). Figure 1 shows the ratio chart result of 1–7 exon deletion (Del 1-7).

Regarding the gene dosage, 67% of cases (75/112) had two copies of the active gene. Three copies and one copy of CYP21A2 were found in 7/112 (6.3%) and 5/112 (4.5%) cases, respectively. Zero copies were reported in 24/112 (21.4%), and four copies were reported in only one patient (0.9%). Genotype results of cases with zero copies of the active gene show homozygous LGC or large deletions.

The majority of single nucleotide variants (SNV) were in a homozygous state, 29/112 (25.9%). Heterozygous SNV was found in 11/112 (9.8%) cases. Compound heterozygous SNV and compound homozygous single nucleotide variants were present in 8/112 (7.1%) and 2/112 (1.8%) cases, respectively. The frequency of genotype distribution of CYP21A2 gene variants among cases is presented in Table 1.

**TABLE 1:** The agreement between MLPA and karyotype among studied cases (n = 112).

Karyotype	MLPA		Total
	Male, *n* (%)	Female, *n* (%)	
46XY	32 (97)	1 (3)	33
46XX	7 (8.9)	72 (91.1)	79
Total	39	73	112
Kappa value* (*p* value)	0.837 (<0.001)		

*Kappa value: < 0: less than chance agreement; 0.01–0.2: slight agreement; 0.21–0.4: fair agreement; 0.41–0.6: moderate agreement; 0.61–0.8: substantial agreement; 0.81–0.99: almost perfect.

## Discussion

To the best of our knowledge, the study includes the largest sample size among Arab countries and the first one to use MLPA assay for the diagnosis of copy number variations in a group of 21-OHD CAH children in Egypt. One hundred and twelve consecutive unrelated 21-OHD CAH patients were enrolled in the present study. The results revealed that 89/112 (79.5%) were diagnosed within the first month of life and highlight the necessity of a neonatal screening program for CAH in Egypt.

All cases in the current study had the classical form of 21-OHD. Eighty-eight out of 112 patients (78.6%) were salt wasting (SW) and 24 out of 112 (21.4%) cases had the simple virilizing (SV) form. Fortunately, affected males are fewer than females, as males with the SW form are at higher risk of adrenal crises and may pass away unnoticed without proper diagnosis. In our study, the female-to-male ratio was 2:1. This is in accordance with previous studies that found the female: male ratio was 3:1 (Al-obaidi et al., 2016).

The wrong sex identification of affected females reared as males in 46.8% of cases can be explained by the high prevalence of female ambiguous genitalia. In the present study, all female cases presented with ambiguous genitalia, except for one case that received prenatal treatment. Reassignment of gender to the female sex in cases with 46XX 21OHD CAH is recommended (Freire et al., 2018) and occurred in 36/37 of the genetic females that were misdiagnosed as males. One genetic female only continued as a male after surgical operations at the request of the parents.

One of the potential roles of MLPA is genetic sex identification due to the presence of probes for y and x chromosomes. The results of sex determination by MLPA showed agreement with the karyotyping results, so MLPA can be used for confirmation of karyotyping (Table 1). In cases with atypical genitalia (SW or SV) and CAH cases with a male external phenotype, karyotype is the only way to prove the genetic sex (whether males or virilized females). Ultrasound can detect the presence of Mullerian structures as a guide for the female internal genitalia. However, it is operator dependent, and sometimes, it can be very difficult to visualize the tiny uterus and ovaries in neonates.

Regarding genotyping, the most frequent variant in the present study was I2G, with an allelic frequency of 31%. The second common variant was large deletions (15.45%), and the third most frequent variant was LGC (7.3%) (Table 2). In 2002, Krone et al.’s study showed similar results (Krone et al., 2002). Copy number variants presented in 15% of cases as simple (11.6%) or combined with SNVs. These simple cases may be missed in genetic diagnosis when using commercial molecular techniques that only test for SNVs. Thus, copy number variations also need to be checked in the cohort due to the high frequency of this cohort (Gao et al., 2019).

**TABLE 2:** Genotype frequency among different phenotypes (n = 112).

Variant	Zygosity	Genotype	Phenotype		*n* (%)
			SW, *n* (%)	SV, *n* (%)	
I2G	Hetero	I2G/N	8 (80)	2 (20)	10 (8.9)
	Homo	I2G/I2G	18 (72)	7 (28)	25 (22.3)
I172N	Hetero	I172 N/N	1 (100)	0 (0)	1 (0.9)
	Homo	I172N/I172N	0 (0)	2 (100)	2 (1.8)
F306+T	Homo	F306 + T/F306 + T	2 (100)	0 (0)	2 (1.8)
LGC (1–3 exon)	Hetero	LGC/N	1 (100)	0 (0)	1 (0.9)
	Homo	LGC/LGC	3 (100)	0 (0)	3 (2.7)
1–3 exon Del	Homo	Del 1–3/Del 1–3	1 (100)	0 (0)	1 (0.9)
1–7 exon Del	Homo	Del 1–7/Del 1–7	4 (100)	0 (0)	4 (3.6)
-113 SNP, I2G	Hetero	(-113 SNP, I2G)/N or^*^ -113 SNP/I2G	1 (100)	0 (0)	1 (0.9)
	Homo	(-113 SNP, I2G)/(-113 SNP, I2G)	2 (100)	0 (0)	2 (1.8)
I2G, 8bp Del	Hetero	(I2G, 8bp Del)/N or* I2G/8bp Del	2 (100)	0 (0)	2 (1.8)
I2G, I172N	Hetero	(I2G, I172N)/N or* I2G/I172N	1 (100)	0 (0)	1 (0.9)
I2G, E6^†^	Hetero	(I2G, E6)/N or* I2G/E6	2 (100)	0 (0)	2 (1.8)
I2G, F306+T	Hetero	(I2G, F306 + T)/N or* I2G/F306 + T	1 (100)	0 (0)	1 (0.9)
I172N, F306+T	Hetero	(I172N, F306 + T)/N or* I172N/F306 + T	0 (0)	1 (100)	1 (0.9)
LGC (1–3 exon), I2G	Hetero	LGC/I2G	2 (100)	0 (0)	2 (1.8)
LGC (1–4 exon), I2G	Hetero	LGC/I2G	1 (100)	0 (0)	1 (0.9)
1–3 exon Del, I2G	Hetero	Del 1–3/I2G	1 (100)	0 (0)	1 (0.9)
LGC (1–3 exon), 1–3 exon Del	Hetero	LGC/Del 1–3	4 (100)	0 (0)	4 (3.6)
LGC (1–3 exon), 1–7 exon Del	Hetero	LGC/Del 1–7	0 (0)	1 (100)	1 (0.9)
LGC (1–7 exon), 1–7 exon Del	Hetero	LGC/Del 1–7	1 (100)	0 (0)	1 (0.9)
LGC (1–4 exon), 30-KB Del (CH-7)	Hetero	LGC/Del	1 (100)	0 (0)	1 (0.9)
30-KB Del (CH-1)	Homo	Del/Del	6 (100)	0 (0)	6 (5.4)
Large gene Del (CAH-X CH-1)	Homo	Del^a^/Del^a^	3 (100)	0 (0)	3 (2.7)
No identified variant	Homo	N/N	22 (66.7)	11 (33.3)	33 (29.5)
Total			88	24	112

LGC, large gene conversion; Del, deletion; N, no identified variant; Homo, homozygous; Hetero, heterozygous; CH-1, chimeric gene produced by 30-KB, Del extending from exon 4 of the *CYP21A1P* pseudogene to exon 3 of the active *CYP21A2* gene; CH-7, chimeric gene produced by 30-KB, Del extending from exon 7 of the *CYP21A1P* pseudogene to exon 6 of the active *CYP21A2* gene; Del^a^, large gene deletion from the *CYP21A2* gene extending to exon 35 of the *TNXB* gene.

†E6: exon 6 cluster variants (V237E and M239K), SW: salt wasting, SV: simple virilizing.

*Compound heterozygous variants may be in *cis* or *trans* configuration and needs further investigations for both parents.

New et al. highlighted the high frequency of homozygous I2G variants in the Middle Eastern population (New et al., 2013). The I2G mutation presented in 31% of cases alone, or combined with other point mutations (9%) or with copy number variations (LGC or deletions) (3.5%). The E6 cluster variants (p.V237E, p.M239K) showed an allele frequency of 0.86%. The homozygous I2G (as the common mutation) and compound heterozygous for I2G and E6 (the least frequent point mutation) mutations cause the same disease phenotype (SW). The genotype frequency among different phenotypes in the studied cases is summarized in Supplementary Table S1.

The lack of identified variants in the present study highlights the need for further genetic studies to detect such a discrepancy between the genotype and phenotype. This may be explained by the presence of common variants of the CYP21A2 gene that were not detected by MLPA probes of probemix P050-C1 CAH as a mutation, P.Q318X, in addition to rare variants that were not tested. Moreover, variants within the promoter region of the gene may be present.

Sequencing is essential to optimize molecular diagnosis in CAH patients. It is considered the only available method that allows for a 100% detection rate for all single nucleotide variants of CYP21A2, either common or rare. Direct sequencing in combination with MLPA offers the highest diagnostic information (Khattab et al., 2016). The gene dosage of the active gene and pseudogene reported in the current study using the MLPA technique showed the CYP21A2 copy number was zero in 24/112 (21.4%). Three copies were found in 7 of the 112 patients (6.3%). To our knowledge, no studies investigate the clinical importance of pseudogene copy number role in the CAH. Pseudogene dosage analysis is important in the differentiation between large deletions and LGC and to avoid misinterpretation of the wild-type sequence of a pseudogene as a duplication of CYP21A2. The present study results showed three copies of CYP21A1P in 21 out of 112 patients (18.8%) and four copies in 4 out of112 (3.6%).

An added diagnostic potential of using the MLPA technique is that the pseudogene analysis was performed simultaneously with CYP21A2 analysis, so this can help to detect the extent of deletion and different forms of chimeric genes. In the present study, a 30-KB Del-produced chimeric gene (CH-1) was detected in 6/112 cases and a chimeric gene (CH-7) in one case. Coeli-Lacchini et al. conducted a study using the MLPA method. The results revealed a 30-kB Del in 3/90 (3.3%) and a complete CYP21A2 deletion in one of the patients (Coeli-Lacchini et al., 2013).

Deletions typically extend approximately 30 kb and have their breakpoint somewhere between exons 3 and 8 of the CYP21A1P pseudogene and ending somewhere at the corresponding point in CYP21A2, thus yielding a single remaining gene in which the 5′ end corresponds to the CYP21A1P pseudogene and the 3′ end corresponds to the CYP21A2 gene. To date, nine types of CYP21A1P/CYP21A2 chimera (CH1–CH9) with different junction sites have been identified (New et al., 2013). Furthermore, loss of MLPA probes specific to the coding regions of the CYP21A2 gene, which constituted loss of exons 1–3, is Del 1–3, loss of exons 1–6 is Del 1–6, and loss of exons 1–8 is Del 1–8 variants in CYP21A2 (Balraj et al., 2013). The classic TNXA/TNXB chimeric gene is a 120 bp deletion at the boundary of exon 35 and intron 35 (CAH-X CH-1). The CYP21A2 gene is completely deleted and replaced by the CYP21A1P pseudogene (Merke et al., 2013). The 148 and 318 nucleotide (nt) probes of TNXB exon 35 of the MLPA probemix (P050-C1 CAH) are located within the 121 nt sequence that is absent in the TNXA pseudogene. Using the MLPA technique, the prevalence of large deletions and large gene conversions in the present study was found in 29/112 (25.9%) patients with the SW phenotype. The results were in agreement with the work of Ben Charfeddine et al., 2012, who reported gene deletion/conversion in 11/50 Tunisian patients (22%) (Kharrat et al., 2004; Ben Charfeddine et al., 2012; Chi et al., 2019; Umaña-Calderón et al., 2021).

**FIGURE 2:**
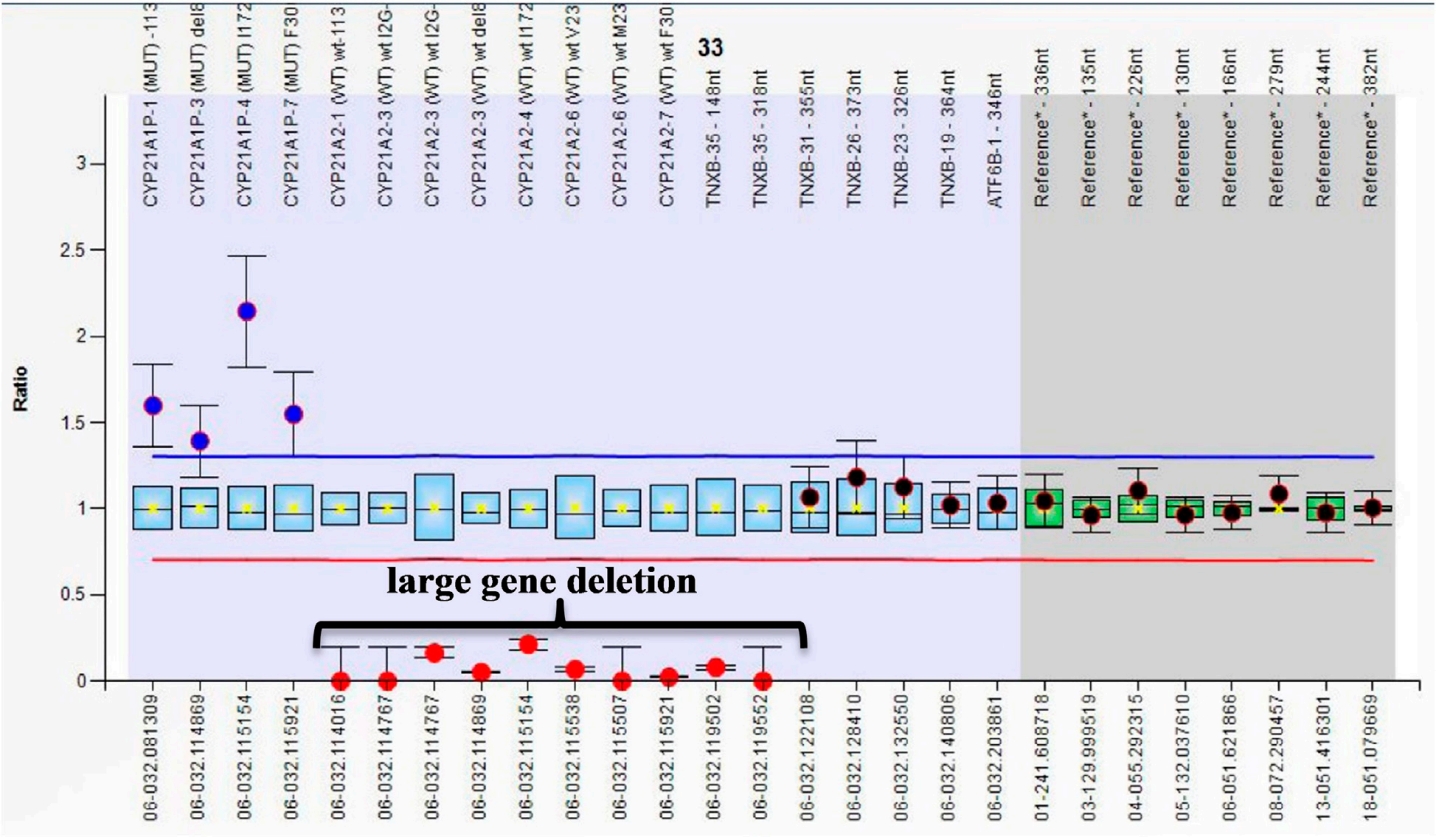
Ratio chart showing homozygous large gene deletion from CYP21A2 extending to exon 35 of TNXB which produced chimeric gene TNXA/TNXB (CAH-X CH-1).

The present study is the first to report large gene Del in Egyptian CAH patients, extending to exon 35 of the TNXB gene that produced a chimeric gene (CAH-X CH-1) in 3/112 patients. All cases with large gene deletions presented with the SW form (Figure 2). This molecular diagnosis coincides with Ehlers–Danlos syndrome (EDS). These results highlight the importance of a complete clinical evaluation for CAH children. Clinical evaluation for connective tissue dysplasia should be routinely performed in CAH patients, especially those harboring a CYP21A2 deletion (Merke et al., 2013,) as CAH-X recently considered a subtype of CAH (Lao and Merke, 2021; Miller, 2021). Merke et al. reported CAH-X syndrome in 7% of CAH patients. Patients with CAH-X were more likely to have joint hypermobility, chronic joint pain, multiple joint dislocations, and structural cardiac valves.

## Conclusion

Early genetic sex assignment, genetic diagnosis, and phenotype prediction are important in the early treatment of CAH patients. Due to the sophisticated mechanisms of genetic variations in the CYP21A2 gene, there is an urgent need to use MLPA as a multipotent and cost-effective laboratory routine tool for the molecular diagnosis of 21-OHD CAH in countries with a high frequency of CAH for proper diagnosis of cases. Recommendations for tailoring the panel of MLPA probe mix to include missed important variants will add to its potent diagnostic effect.

## Data Availability

The original contributions presented in the study are included in the article/Supplementary Material, further inquiries can be directed to the corresponding author.

## References

[B1] Al-obaidi R. G. Y. Al-musawi B. M. S. Al-zubaidi M. A. K. Oberkanins C. Németh S. Al-obaidi Y. G. Y. (2016). Molecular Analysis of CYP21A2 Gene Mutations Among Iraqi Patients with Congenital Adrenal Hyperplasia. Enzyme Res. 2016, 1–7. 10.1155/2016/9040616 PMC5061977 27777794

[B2] Alshabab L. I. S. Alebrahem A. Kaddoura A. Al-Fahoum S. (2015). Congenital Adrenal Hyperplasia Due to 21-hydroxylase Deficiency: A Five-Year Retrospective Study in the Children's Hospital of Damascus, Syria. Qatar Med. J. 2015, 11. 10.5339/qmj.2015.11 26535179 PMC4614327

[B3] Balraj P. Lim P. G. Sidek H. Wu L. L. Khoo A. S. (2013). Mutational Characterization of Congenital Adrenal Hyperplasia Due to 21-hydroxylase Deficiency in Malaysia. J. Endocrinol. Invest. 36, 366–374. 10.3275/8648 23027774

[B4] Ben Charfeddine I. Riepe F. G. Clauser E. Ayedi A. Makni S. Sfar M. T. (2012). Steroid 21-hydroxylase Gene Mutational Spectrum in 50 Tunisian Patients: Characterization of Three Novel Polymorphisms. Gene 507, 20–26. 10.1016/j.gene.2012.07.027 22841790

[B5] Blaskó B. Bánlaki Z. Gyapay G. Pozsonyi É. Sasvári-Székely M. Rajczy K. (2009). Linkage Analysis of the C4A/C4B Copy Number Variation and Polymorphisms of the Adjacent Steroid 21-hydroxylase Gene in a Healthy Population. Mol. Immunol. 46, 2623–2629. 10.1016/j.molimm.2009.04.033 19505723

[B6] Chan A. O. K. But W. M. Ng K. L. Wong L. M. Lam Y. Y. Tiu S. C. (2011). Molecular Analysis of Congenital Adrenal Hyperplasia Due to 21-hydroxylase Deficiency in Hong Kong Chinese Patients. Steroids 76, 1057–1062. 10.1016/j.steroids.2011.04.010 21570420

[B7] Chen W. Xu Z. Sullivan A. Finkielstain G. P. Van Ryzin C. Merke D. P. (2012). Junction Site Analysis of Chimeric CYP21A1P/CYP21A2 Genes in 21-hydroxylase Deficiency. Clin. Chem. 58, 421–430. 10.1373/clinchem.2011.174037 22156666 PMC5576027

[B8] Chi D. V. Tran T. H. Nguyen D. H. Luong L. H. Le P. T. Ta M. H. (2019). Novel Variants of CYP21A2 in Vietnamese Patients with Congenital Adrenal Hyperplasia. Mol. Genet. Genomic Med. 7, e623. 10.1002/mgg3.623 30816000 PMC6503067

[B9] Choi J.-H. Kim G.-H. Yoo H.-W. (2016). Recent Advances in Biochemical and Molecular Analysis of Congenital Adrenal Hyperplasia Due to 21-hydroxylase Deficiency. Ann. Pediatr. Endocrinol. Metab. 21, 1–6. 10.6065/apem.2016.21.1.1 27104172 PMC4835555

[B10] Coeli-Lacchini F. B. Turatti W. Elias P. C. L. Elias L. L. K. Martinelli C. E. Moreira A. C. (2013). A Rational, Non-radioactive Strategy for the Molecular Diagnosis of Congenital Adrenal Hyperplasia Due to 21-hydroxylase Deficiency. Gene 526, 239–245. 10.1016/j.gene.2013.03.082 23570880

[B11] Concolino P. Mello E. Toscano V. Ameglio F. Zuppi C. Capoluongo E. (2009). Multiplex Ligation-dependent Probe Amplification (MLPA) Assay for the Detection of CYP21A2 Gene Deletions/duplications in Congenital Adrenal Hyperplasia: First Technical Report. Clinica Chim. Acta 402, 164–170. 10.1016/j.cca.2009.01.008 19263525

[B12] Daae E. Feragen K. B. Nermoen I. Falhammar H. (2018). Psychological Adjustment, Quality of Life, and Self-Perceptions of Reproductive Health in Males with Congenital Adrenal Hyperplasia: a Systematic Review. Endocrine 62, 3–13. 10.1007/s12020-018-1723-0 30128958 PMC6153586

[B13] den Dunnen J. T. Dalgleish R. Maglott D. R. Hart R. K. Greenblatt M. S. McGowan-Jordan J. HGVS Recommendations for the Description of Sequence Variants: 2016 Update. Hum. Mutat. 2016;37:564–569. 10.1002/humu.22981 26931183

[B14] Erlandson A. Appelqvist F. Enerbäck C. (2008). Epigenetic Mutations in CDKN2A in Western Swedish Families with Hereditary Malignant Melanoma. Mol. Med. Rep. 1, 89–91. 21479383

[B15] Freire A. V. Grinspon R. P. Rey R. A. (2018). Importance of Serum Testicular Protein Hormone Measurement in the Assessment of Disorders of Sex Development. Sex. Dev. 12, 30–40. 10.1159/000479572 28850950

[B16] Gao Y. J. Yu B. Q. Lu L. Wu X. Y. Mao J. F. Wang X. (2019). Analysis of Copy Number Variation of CYP21A2 Gene and the Type of CYP21A1P/CYP21A2 Fused Gene in Patients with 21-hydroxylase Deficiency. Zhonghua Yi Xue Za Zhi 99, 3765–3769. 10.3760/cma.j.issn.0376-2491.2019.48.002 31874511

[B17] Guerrero-Fernández J. Azcona San Julián C. Barreiro Conde J. Bermúdez de la Vega J. A. Carcavilla Urquí A. Castaño González L. A. (2018). Guía de actuación en las anomalías de la diferenciación sexual (ADS)/desarrollo sexual diferente (DSD). Anales de Pediatría 89, e1–315. 10.1016/j.anpedi.2018.06.009 30033107

[B18] Huynh T. McGown I. Cowley D. Nyunt O. Leong G. M. Harris M. (2009). The Clinical and Biochemical Spectrum of Congenital Adrenal Hyperplasia Secondary to 21-hydroxylase Deficiency. Clin. Biochem. Rev. 30, 75–86. 19565027 PMC2702216

[B19] Kharrat M. Tardy V. M’Rad R. Maazoul F. Jemaa L. B. Refaï M. (2004). Molecular Genetic Analysis of Tunisian Patients with a Classic Form of 21-hydroxylase Deficiency: Identification of Four Novel Mutations and High Prevalence of Q318X Mutation. J. Clin. Endocrinol. Metab. 89, 368–374. 10.1210/jc.2003-031056 14715874

[B20] Khattab A. Yuen T. Al-Malki S. Yau M. Kazmi D. Sun L. (2016). A rareCYP21A2mutation in a Congenital Adrenal Hyperplasia kindred Displaying Genotype-Phenotype Nonconcordance. Ann. N.Y. Acad. Sci. 1364, 5–10. 10.1111/nyas.12864 26291314 PMC4761329

[B21] Krone N. Braun A. Weinert S. Peter M. Roscher A. A. Partsch C.-J. (2002). Multiplex Minisequencing of the 21-hydroxylase Gene as a Rapid Strategy to Confirm Congenital Adrenal Hyperplasia. Clin. Chem. 48, 818–825. 10.1093/clinchem/48.6.818 12028996

[B22] Kutney K. Konczal L. Kaminski B. Uli N. Challenges in the Diagnosis and Management of Disorders of Sex Development. Birth Defects Res. C Embryo Today 2016;108:293–308. 10.1002/bdrc.21147 28033665

[B23] Lao Q. Merke D. P. (2021). Molecular Genetic Testing of Congenital Adrenal Hyperplasia Due to 21-hydroxylase Deficiency Should Include CAH-X Chimeras. Eur. J. Hum. Genet. 29, 1047–1048. 10.1038/s41431-021-00870-5 33824469 PMC8298381

[B24] Lee H. J. Choi J. Hwang T. S. Shong Y. K. Hong S. J. Gong G. (2010). Detection ofBRAFMutations in Thyroid Nodules by Allele-specific PCR Using a Dual Priming Oligonucleotide System. Am. J. Clin. Pathol. 133, 802–808. 10.1309/AJCPO3F2ENKMDTUS 20395530

[B25] Merke D. P. Chen W. Morissette R. Xu Z. Van Ryzin C. Sachdev V. (2013). Tenascin-X Haploinsufficiency Associated with Ehlers-Danlos Syndrome in Patients with Congenital Adrenal Hyperplasia. J. Clin. Endocrinol. Metab. 98, E379–E387. 10.1210/jc.2012-3148 23284009 PMC3565116

[B26] Miller W. L. (2021). Tenascin-X-Discovery and Early Research. Front. Immunol. 11, 612497. 10.3389/fimmu.2020.612497 33505400 PMC7829301

[B27] Musa N. Asem N. Basyony S. Fawaz L. (2020). Assessment of Health-Related Quality of Life in Egyptian Children and Adolescents with Congenital Adrenal Hyperplasia. J. Pediatr. Endocrinol. Metab. 33, 295–304. 10.1515/jpem-2019-0345 32004147

[B28] New M. I. Abraham M. Gonzalez B. Dumic M. Razzaghy-Azar M. Chitayat D. (2013). Genotype-phenotype Correlation in 1,507 Families with Congenital Adrenal Hyperplasia Owing to 21-hydroxylase Deficiency. Proc. Natl. Acad. Sci. 110, 2611–2616. 10.1073/pnas.1300057110 23359698 PMC3574953

[B29] Nimkarn S. Gangishetti P. K. Yau M. New M. I. (1993). in 21-Hydroxylase-Deficient Congenital Adrenal Hyperplasia. Adam M. P. Ardinger H. H. Pagon R. A. Wallace S. E. Bean L. J. H. Stephens K.. Editors (Seattle (WA.

[B30] Rabbani B. Mahdieh N. Haghi Ashtiani M. T. Akbari M. T. Rabbani A. (2011). Molecular Diagnosis of Congenital Adrenal Hyperplasia in Iran: Focusing on CYP21A2 Gene. Iran J. Pediatr. 21, 139–150. 23056780 PMC3446151

[B31] Savanelli A. Alicchio F. Esposito C. De Marco M. Settimi A. (2008). A Modified Approach for Feminizing Genitoplasty. World J. Urol. 26, 517–520. 10.1007/s00345-008-0298-4 18594825

[B32] Speiser P. W. Arlt W. Auchus R. J. Baskin L. S. Conway G. S. Merke D. P. (2018). Congenital Adrenal Hyperplasia Due to Steroid 21-Hydroxylase Deficiency: An Endocrine Society* Clinical Practice Guideline. J. Clin. Endocrinol. Metab. 103, 4043–4088. 10.1210/jc.2018-01865 30272171 PMC6456929

[B33] Tayel S. M. Ismael H. Kandil H. Abd Rabuh A. R. Sallam H. (2011). Congenital Adrenal Hyperplasia in Alexandria, Egypt: A High Prevalence Justifying the Need for a Community-Based Newborn Screening Program. J. Trop. Pediatr. 57, 232–234. 10.1093/tropej/fmq064 20615895

[B34] Temtamy S. Aglan M. (2012). Consanguinity and Genetic Disorders in Egypt. Middle East. J. Med. Genet. 1. 10.1097/01.mxe.0000407744.14663.d8

[B35] Toraman B. Ökten A. Kalay E. Karagüzel G. Dinçer T. Açıkgöz E. G. (2013). Investigation of CYP21A2 Mutations in Turkish Patients with 21-hydroxylase Deficiency and a Novel Founder Mutation. Gene 513, 202–208. 10.1016/j.gene.2012.10.059 23142378

[B36] Tsai M.-C. Chou Y.-Y. Lin S.-J. Tsai L.-P. (2012). A Novel SRD5A2 Mutation in a Taiwanese Newborn with Ambiguous Genitalia. Kaohsiung J. Med. Sci. 28, 231–235. 10.1016/j.kjms.2011.10.011 22453073 PMC11916018

[B37] Umaña-Calderón A. Acuña-Navas M. J. Alvarado D. Jiménez M. Cavallo-Aita F. (2021). CYP21A2 Mutations in Pediatric Patients with Congenital Adrenal Hyperplasia in Costa Rica. Mol. Genet. Metab. Rep. 27, 100728. 10.1016/j.ymgmr.2021.100728 33604243 PMC7875833

[B38] Veldhuisen B. van der Schoot C. E. de Haas M. (2015). Multiplex Ligation-dependent Probe Amplification (MLPA) Assay for Blood Group Genotyping, Copy Number Quantification, and Analysis of RH Variants. Immunohematology 31, 58–61. 26495890

[B39] Vrzalová Z. Hrubá Z. Hrabincová E. S. Vrábelová S. Votava F. Koloušková S. (2011). Chimeric CYP21A1P/CYP21A2 Genes Identified in Czech Patients with Congenital Adrenal Hyperplasia. Eur. J. Med. Genet. 54, 112–117. 10.1016/j.ejmg.2010.10.005 20970527

[B40] Wisniewski A. B. (2017). Psychosocial Implications of Disorders of Sex Development Treatment for Parents. Curr. Opin. Urol. 27, 11–13. 10.1097/MOU.0000000000000344 27584026 PMC5283739

[B41] Xu Z. Chen W. Merke D. P. McDonnell N. B. (2013). Comprehensive Mutation Analysis of the CYP21A2 Gene. J. Mol. Diagn. 15, 745–753. 10.1016/j.jmoldx.2013.06.001 24071710 PMC5803549

