# Supplementary material for: Copy Number Variations in Genetic Diagnosis of Congenital Adrenal Hyperplasia Children
Source: Front Genet. 2022 Mar 2;13:785570. doi: 10.3389/fgene.2022.785570 (PMC8924405; doi:10.3389/fgene.2022.785570)
Supplement: Supplementary file 1 [file Table2.DOCX]

**Table (S1):** Genotype frequency among different phenotypes in previous studies

| **Variant** | **Zygosity** | **Genotype** | no. of studied cases | **Phenotype** | | | ***n* (%)** | ***References*** |
| --- | --- | --- | --- | --- | --- | --- | --- | --- |
|  |  |  |  | SW  n (%) | SV  n (%) | **NC**  **n (%)** |  |  |
| **I2G** | Hetero | I2G/N | 155  52  97  14  55 | 18 (90)  1 (100)  4 (100)  1 (100)  2 (33) | 2 (10)  0 (0)  0 (0)  0 (0)  3 (50) | 0 (0)  0 (0)  0 (0)  0 (0)  1 (17) | 20 (12.9)  1 (1.9)  4 (4.1)  1 (7.1)  6 (10.9) | Krone et al., 2000[1]  Baş et al. 2009[2]  Balraj et al., 2013[3]  Elmougy et al. 2018[4]  Espinosa Reyes et al., 2020[5] |
|  | Homo | I2G/I2G | 51  52  213  44  1507  204  72  55  628 | 7 (100)  3 (50)  8 (89)  4 (80)  143(92)  27 (88)  9 (90)  4 (67)  10 (45) | 0 (0)  3 (50)  1 (11)  1 (20)  11 (7)  2 (6)  1 (10)  2 (33)  12 (55) | 0 (0)  0 (0)  0 (0)  0 (0)  1 (1)  2 (6)  0 (0)  0 (0)  0 (0) | 7 (13.7)  6 (11.5)  9 (4.2)  5 (11.4)  155 (10.3)  31 (15.4)  10 (13.9)  6 (10.9)  22 (3.5) | Kharrat et al., 2004[6]  Baş et al. 2009[2]  Finkielstain et al., 2011[7]  Rabbani et al., 2011[8]  New et al., 2013[9]  Chi et al., 2019[10]  Xu et al., 2019[11]  Espinosa Reyes et al., 2020[5]  Fernandez et al., 2020[12] |
| **I172N** | Hetero | I172N/N | 97 | 1 (100) | 0 (0) | 0 (0) | 1 (1) | Balraj et al., 2013[3] |
|  | Homo | I172N/I172N | 155  51  52  213  44  1507  204  72  55  628 | 0 (0)  0 (0)  0 (0)  0 (0)  0 (0)  2 (9)  1 (11)  1 (20)  1 (100)  1 (10) | 7 (100)  5 (100)  2 (100)  3 (75)  2 (100)  21(91)  8 (89)  1 (20)  0 (0)  9 (90) | 0 (0)  0 (0)  0 (0)  1 (25)  0 (0)  0 (0)  0 (0)  3 (60)  0 (0)  0 (0) | 7 (4.5)  5 (9.8)  2 (3.8)  4 (1.9)  2 (4.5)  23 (1.5)  9 (4.4)  5 (6.9)  1 (1.8)  10 (1.6) | Krone et al., 2000[1]  Kharrat et al., 2004[6]  Baş et al. 2009[2]  Finkielstain et al., 2011[13]  Rabbani et al., 2012[8]  New et al., 2013[9]  Chi et al., 2019[10]  Xu et al., 2019[14]  Espinosa Reyes et al., 2020[5]  Fernandez et al., 2020[12] |
| **1-3 exon Del** | Homo | Del 1-3/Del 1-3 | 97  204 | 1 (100)  11 (100) | 0 (0)  0 (0) | 0 (0)  0 (0) | 1 (1)  11 (5.5) | Balraj et al., 2013[3]  Chi et al., 2019[10] |
| **I2G, 8bp Del** | Hetero | (I2G, 8bp Del)/N or* I2G/8bp Del | 155  52  213  1507  72  55  628 | 2 (67)  1 (100)  1 (100)  10 (72)  1 (100)  0 (0)  0 (0) | 1 (33)  0 (0)  0 (0)  3 (21)  0 (0)  1(100)  1(100) | 0 (0)  0 (0)  0 (0)  1 (7)  0 (0)  0 (0)  0 (0) | 3 (1.9)  1 (1.9)  1 (0.5)  14 (0.9)  1 (1.4)  1 (1.8)  1 (0.2) | Krone et al., 2000[1]  Baş et al. 2009[2]  Finkielstain et al., 2011[13]  New et al., 2013[9]  Xu et al., 2019[14]  Espinosa Reyes et al., 2020[5]  Fernandez et al., 2020[12] |
| **I2G, I172N** | Hetero | (I2G, I172N)/N or* I2G/I172N | 155  213  97  1507  204  72  628 | 5 (26)  1 (10)  1 (25)  13 (26)  0 (0)  0 (0)  1 (5) | 14 (74)  8 (80)  3 (75)  36 (72)  4 (100)  4 (80)  19 (95) | 0 (0)  1 (10)  0 (0)  1 (2)  0 (0)  1 (20)  0 (0) | 19 (12.3)  10 (4.7)  4 (4.1)  50 (3.3)  4 (2)  5 (6.9)  20 (3.2) | Krone et al., 2000[1]  Finkielstain et al., 2011[13]  Balraj et al., 2013[3]  New et al., 2013[9]  Chi et al., 2019[10]  Xu et al., 2019[14]  Fernandez et al., 2020[12] |
| **I2G, E6^†^** | Hetero | (I2G, E6)/N or* I2G/E6 | 155  213  97  1507  628 | 1 (100)  1 (100)  1 (100)  14 (100)  1 (100) | 0 (0)  0 (0)  0 (0)  0 (0)  0 (0) | 0 (0)  0 (0)  0 (0)  0 (0)  0 (0) | 1 (0.6)  1 (0.5)  1 (1)  14 (0.9)  1 (0.2) | Krone et al., 2000[1]  Finkielstain et al., 2011[13]  Balraj et al., 2013[3]  New et al., 2013[9]  Fernandez et al., 2020[12] |
| **I2G, F306+T** | Hetero | (I2G, F306+T)/N or* I2G/F306+T | 155  97  628 | 1 (100)  1 (100)  1 (100) | 0 (0)  0 (0)  0 (0) | 0 (0)  0 (0)  0 (0) | 1 (0.6)  1 (1)  1 (0.2) | Krone et al., 2000[1]  Balraj et al., 2013[3]  Fernandez et al., 2020[12] |
| **LGC (1-3 exon), I2G** | Hetero | LGC/I2G | 99 | 1 (100) | 0 (0) | 0 (0) | 1 (1) | Coeli-Lacchini et al., 2013[15] |
| **LGC (1-4 exon), I2G** | Hetero | LGC/I2G | 99 | 1 (100) | 0 (0) | 0 (0) | 1 (1) | Coeli-Lacchini et al., 2013[14] |
| **1-3 exon Del, I2G** | Hetero | Del 1-3/I2G | 97 | 6 (100) | 0 (0) | 0 (0) | 6 (6.2) | Balraj et al., 2013[3] |
| **30-KB Del (CH-1)** | Homo | Del/Del | 44 | 3 (75) | 1 (25) | 0 (0) | 4 (9.1) | Rabbani et al., 2012[8] |
| **No identified variant** | Homo | N/N | 51  52  97  58 | 1 (100)  1 (50)  0 (0)  2 (50) | 0 (0)  1 (50)  2 (100)  2 (50) | 0 (0)  0 (0)  0 (0)  0 (0) | 1 (2)  2 (3.8)  2 (2.1)  4 (6.9) | Kharrat et al., 2004[6]  Baş et al. 2009[2]  Balraj et al., 2013[3]  [Umaña-Calderón](https://pubmed.ncbi.nlm.nih.gov/?term=Uma%C3%B1a-Calder%C3%B3n+A&cauthor_id=33604243) et al., 2021[16] |

LGC, large gene conversion; Del, deletion; N, no identified variant; Homo, homozygous; Hetero, heterozygous; CH-1, chimeric gene produced by 30-KB Del extending from exon 4 of *CYP21A1P* pseudogene to exon 3 of active *CYP21A2* gene.

**^†^**E6: Exon 6 cluster variants (V237E, M239K, I236N). SW: salt wasting, SV: simple virilizing.

*Compound heterozygous variants may be in *cis* or *trans* configuration so needs further investigations for both parents.
